# Monitoring of Enterovirus D68 Outbreak in Israel by a Parallel Clinical and Wastewater Based Surveillance

**DOI:** 10.3390/v14051010

**Published:** 2022-05-09

**Authors:** Oran Erster, Itay Bar-Or, Virginia Levy, Rachel Shatzman-Steuerman, Danit Sofer, Leah Weiss, Rinat Vasserman, Ilana S. Fratty, Klil Kestin, Michal Elul, Nofar Levi, Rola Alkrenawi, Ella Mendelson, Michal Mandelboim, Merav Weil

**Affiliations:** 1 Central Virology Laboratory, Ministry of Health, Chaim Sheba Medical Center, Ramat Gan 52621, Israel; itay.baror@sheba.health.gov.il (I.B.-O.); virgini.levy@sheba.health.gov.il (V.L.); danit.sofer@sheba.health.gov.il (D.S.); leah.weiss@sheba.health.gov.il (L.W.); rinat.vasserman@sheba.health.gov.il (R.V.); klil.kestin@sheba.health.gov.il (K.K.); michal.elul@sheba.health.gov.il (M.E.); nofar.levi@sheba.health.gov.il (N.L.); rola.grenawe@sheba.health.gov.il (R.A.); ella.mendelson@sheba.health.gov.il (E.M.); michal.mandelboim@sheba.health.gov.il (M.M.); 2 School of Public Health, Sackler Faculty of Medicine, Tel-Aviv University, Tel-Aviv 69978, Israel; rachel.steuerman@clalit.org.il; 3 Pediatric Infectious Diseases Unit, Chaim Sheba Medical Center, Ramat Gan 52621, Israel; 4 Israel Center for Disease Control, Israel Ministry of Health, Sheba Medical Center, Tel-Hashomer, Ramat Gan 52621, Israel; ilana.fratty@sheba.health.gov.il

**Keywords:** enterovirus D68, quantitative PCR, wastewater-based epidemiology, clinical surveillance

## Abstract

Enterovirus D68 (EVD68) was recently identified as an important cause of respiratory illness and acute flaccid myelitis (AFM), mostly in children. Here, we examined 472 pediatric patients diagnosed with severe respiratory illness and screened for EVD68 between April and October 2021. In parallel, samples collected from a wastewater treatment plant (WWTP) covering the residential area of the hospitalized patients were also tested for EVD68. Of the 472 clinical samples evaluated, 33 (7%) patients were positive for EVD68 RNA. All wastewater samples were positive for EVD68, with varying viral genome copy loads. Calculated EVD68 genome copies increased from the end of May until July 2021 and dramatically decreased at the beginning of August. A similar trend was observed in both clinical and wastewater samples during the period tested. Sequence analysis of EVD68-positive samples indicated that all samples originated from the same branch of subclade B3. This study is the first to use wastewater-based epidemiology (WBE) to monitor EVD68 dynamics by quantitative detection and shows a clear correlation with clinically diagnosed cases. These findings highlight the potential of WBE as an important tool for continuous surveillance of EVD68 and other enteroviruses.

## 1. Introduction

Human enterovirus D68 (EV-D68) belongs to Enterovirus D, a species in the Picornaviridae family. EV-D68 is acid-labile and has a phenotype closely related to human rhinoviruses [1]. Unlike other types of enteroviruses, EV-D68 has been associated mainly with acute respiratory infection with clinical presentations ranging from mild to severe disease requiring intensive care treatment. EV-D68 also has been reported to cause acute flaccid myelitis (AFM) [2]. Since 2014, an increase in worldwide EV-D68 positive cases has been observed, with a biennial pattern, following the expansion of clinical surveillance systems [3,4] and improved availability of molecular testing. However, the surveillance system for EVD68 is still limited and voluntary worldwide. In Israel, EVD68 is currently not monitored routinely; thus, its circulation in Israel is not well understood.

Wastewater-based epidemiology (WBE) is a useful tool to monitor the circulation of enteric viruses and their variants, such as Poliovirus [5]. Since the beginning of the COVID-19 pandemic, the WBE approach has been applied to study SARS-CoV-2 epidemiology and gradually has become more widely used worldwide [6].

EVD68 is primarily detected in respiratory tract tissues; however, it has been shown that it can also be detected in the gastrointestinal tract [7]. Moreover, several studies demonstrated that EVD68 could be detected in wastewater samples with relevance to infections in the clinical setting [8,9,10]. In this study, we demonstrated for the first time the detection of unusual EVD68 circulation in wastewater, using quantitative RT-PCR (qPCR). We showed that wastewater surveillance can serve as an early warning system for EVD68 outbreaks with respect to clinical EVD68 infections diagnosed in central Israel.

## 2. Materials and Methods

### 2.1. Clinical Sample Collection and Processing

A retrospective study was performed using respiratory clinical samples (nasopharyngeal aspirates (NPAs), nasal swabs and bronchoalveolar lavage) collected from 472 hospitalized pediatric patients (aged < 18 years) mostly suffering from respiratory illnesses between April and October 2021. The rationale for testing for EVD68 was either respiratory or neurological symptoms or continuous fever, with negative diagnosis of other relevant pathogens. The nasopharyngeal samples of the hospitalized patients were extracted using STARMag universal kit, following the manufacturer’s instructions. The RT-PCR test for identifying the respiratory viruses was conducted with the AllplexTM RV7 essential assay from Seegene [11]. All clinical samples that were examined in this study tested negative for influenza A, B, hMPV, rhinovirus, adenovirus, parainfluenza-3 and RSV by qPCR.

### 2.2. Wastewater Sample Preparation

Wastewater samples were collected from the Tel-Aviv region wastewater treatment plant (WWTP) once a week between April and October 2021 (n = 29 samples). This WWTP covers the residential area of the hospitalized patients. Data from weeks 20, 25 and 36 were not available due to sampling process malfunctions. Two hundred and fifty mL of untreated wastewater were collected every 30 min for 24 h by external automatic composite samplers. Following collection, samples were transported to the laboratory at 4 °C. Viral particles were concentrated from each wastewater sample as previously described [12]. Extracted NA were eluted in 55 μL elution buffer and stored at −70 °C for further use.

### 2.3. Detection of EV-D68 RNA by Quantitative PCR (RT-qPCR)

Detection of EVD68 was performed using a duplex assay including the reaction developed by Ikuse et al. [13], and a reaction that detects the presence of MS-2 phage RNA [14], which was used as a spiked control. Briefly, two sets of primers and fluorescent probes were used, and the mix was prepared according to the sample type. For clinical samples, the SensiFast mix was used [15]. For wastewater samples, the inhibitor-resistant Reliance mix was used [16]. The details of the assembly and thermal profiles for each reaction type are shown in Supplementary Tables S1 and S2. All reactions were run in a CFX-96 thermal cycler [16] and analyzed using the accompanying software.

### 2.4. Generation of EVD68 RNA Standard and Calculation of EVD68 RNA Copies

In order to determine the sensitivity of the duplex reaction and quantify the PCR results, RNA standards were synthesized and used for analytic limit of detection analysis. The region spanning the qPCR target sequence was amplified using the following primers: pT7_EVD68_2360Fwd and 2985 Rev. The primer sequences were as follows: pT7_EVD68_2360Fwd: 5′-TAATACGACTCACTATAGGGTCCCTCAGRTTAATGAGAGA-3′ and 2985 Rev: 5′-CACATAAAAGGTATRGTCATTC-3′. Bolded letters represent the minimal T7 promoter. The forward primer included the minimal T7 promoter (in bold letters), which was then used for in vitro transcription (IVT) of the PCR product. The control fragment was amplified using the reaction detailed in Supplementary Table S3. The amplification reaction was performed using the following thermal profile: 15 min at 45 °C, 2 min at 95 °C, 40 × (10 s at 95 °C, 10 s at 55 °C, 32 s at 72 °C), 5 min at 72 °C. The resulting product was 640 bp long. RNA IVT synthesis was performed using the T7 Megascript kit, according to the manufacturer’s instructions [17]. The resulting RNA product was purified using the PSS MagLEAD system [18], and its concentration was measured using a NanoDrop spectrophotometer [17]. The number of target copies was calculated based on the size of the amplified control standard using the calculator available from SciencePrimer [19]. The length of the standard RNA product was 640 nucleotides. Therefore, the calculated number of copies in 1 ng was 2.77 × 10^9^. This conversion was then used to back-calculate the copies in unknown wastewater samples. For example, the formula that represents the ratio between the target copies and the resulting Cq value in the experiment, shown in Supplementary Figure S1, was Cq = −1.322ln(copies) + 35.999. In order to back-calculate the number of copies in an unknown sample, based on the Cq value, the formula was inverted to the following: Copies = e^(((35.999 − Cq)/1.322)). This procedure was employed in each experiment to calculate the number of target copies in each sample.

### 2.5. EV-D68 VP1 Sequencing and Phylogenetic Analysis

In order to determine the clades of the EV-D68 viruses detected in patients and sewage samples, partial VP1 capsid gene from both sewage and clinical samples was amplified and sequenced as previously described by Ikuse et al. [12]. Sequencing of the amplified PCR products was performed using 3500 ABI Genetic Analyzer [20]. The EV-D68 VP1 sequences from clinical and wastewater samples were deposited in the GenBank with access numbers OM251503 to OM251530. Phylogenetic analyses of the EV-D68 partial VP1 sequences were compared with the EV-D68 reference strains by maximum likelihood analysis using MEGA 7.0 software [21] with 1000 bootstraps.

## 3. Results

### 3.1. Calibration of the Quantitative Duplex EVD68-MS2 Assay

The assay limit of detection for EVD68 RNA was determined using in vitro transcribed RNA (IVT) standard in serial dilutions. In order to verify that the IVT standard was consistent with the whole genome of EVD68 RNA, the calibration assay was performed using a combination of the IVT standard serial dilutions and a serially diluted commercial quantified EVD68 RNA [22]. The Cq values obtained from the IVT standard were consistent with those obtained with the commercial whole-genome dilutions, indicating that the IVT standard indeed reflected the actual sensitivity of the assay. The calibration assay successfully detected less than one copy per µL, corresponding to a single copy in the reaction (Supplementary Figure S1).

In order to quantify the viral RNA copies in environmental samples, serial dilutions of standard RNA were tested together with wastewater samples. The formula derived from the standard curve was then used to convert the Cq values obtained for each sample to viral RNA copies, as described in Section 2.4 above.

### 3.2. Characterization and EVD68 Infection and Monitoring of EVD68 RNA in Wastewater Samples

**Figure 1:**
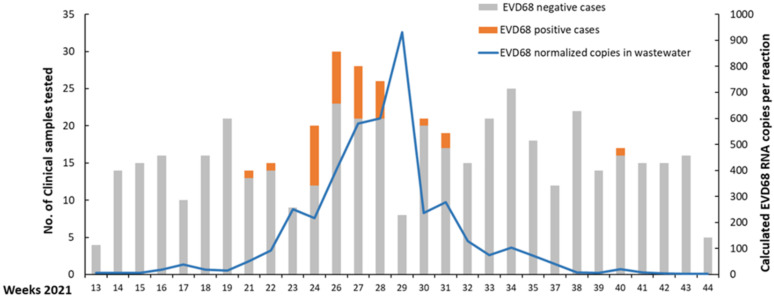
Dynamics of EVD68 circulation in clinical and WW samples. Stacked columns represent the total number of clinical tests for each month, of which the gray column represents the negative and the orange represents the positive. Stacked line represents the calculated number of copies in WW samples, in each week. The left *Y*-axis refers to the number of clinical samples tested in each week. The right *Y*-axis represents the calculated average number of copies per reaction in WW samples in each week.

A total of 472 clinical samples were evaluated, taken from patients ranging in age from 1 month to 14 years old, with an average of 2.6 years. Of these, 33 (7%) were positive for EV-D68 RNA (Table 1). Within the positive samples, 19 (58%) were from males and 14 (42%) from females. Of the 33 positive cases, two presented with fever, 11 had respiratory symptoms, and 18 had both, all of which requiring patient hospitalization. The most common underlying disease was asthma. Five patients were co-infected with other pathogens, and two did not have respiratory symptoms but suffered from meningitis or encephalitis. Notably, none of the patients was reported to have flaccid paralysis. Complete details on the clinical symptoms of each patient are listed in Table 1. Wastewater (WW) analysis for EVD68 was performed as part of routine surveillance, with the process taking 3–4 days from sample collection to analyzed results. Analysis of the dynamics of clinical cases and the presence of EVD68 RNA in wastewater (WW) during 2021 showed a marked increase in positive cases in weeks 24–28, after which the positive cases declined to zero in week 32, with a single positive case out of 16 samples in week 40. Examination of EVD68 circulation in wastewater showed a marked increase from week 22 to week 29, where the calculated number of viral copies exceeded 900 copies per reaction. In the following weeks, the circulation decreased at approximately the same rate, falling to less than 10 copies per reaction in week 38 (Figure 1).

### 3.3. Phylogenetic Analysis

**Figure 2:**
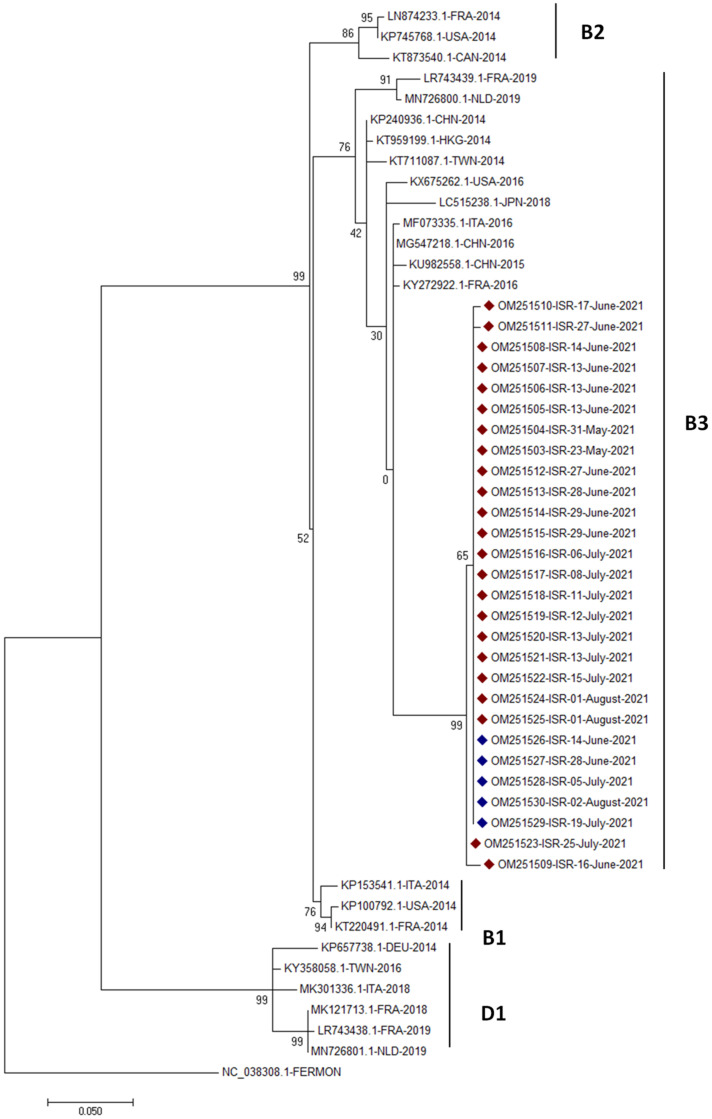
Phylogenetic analysis of EV-D68 sequences obtained in this study. A sequence of 280 nucleotides from EVD68 VP1 gene was amplified and analyzed by constructing a phylogenetic dendrogram together with representing sequences from different strains. The dendrogram was generated using MEGA 7 [21] with 1000 bootstrap replications. Bootstrap values are shown at the branch nodes. The reference strains are each labeled with their accession number, country of origin and year of collection. The samples from Israel were marked in color, as follows: clinical samples in red and sewage samples in blue.

Sequence analysis of 280 nucleotides of the VP1 gene from 28 EV-D68 positive patient and sewage samples showed very close similarity between all samples. The sequences derived from those samples were compared to reference strains that represent a collection of EV-D68 sequences from previously reported world outbreaks during 2014–2020 [23]. The phylogenetic analysis indicated that all local samples were of subclade B3 (Figure 2).

## 4. Discussion

Currently, surveillance of EVD68 is limited and sporadic worldwide. EVD68 surveys were performed on a wide scale in Israel during 2014, with sporadic cases detected [10]. Since 2014, EVD68 has not been monitored routinely in Israel. Therefore, its circulation on a national scale is currently unknown. Here, we performed a retrospective study examining clinical and WW samples collected between April and October 2021. Clinical samples were collected from 472 pediatric patients suffering from respiratory illnesses and were negative for common respiratory viruses. We identified a considerable positive EVD68 rate (20–40%) in these samples from the end of June until mid-July 2021. This upsurge was consistent with the known seasonality of EVD68 [7]. A similar upsurge in EVD68 circulation was reported in September 2021 in Europe and was presumably related to the widespread reopening after COVID-19 lockdowns [24]. During the examined period, the dynamic change in EVD68 circulation in WW samples was similar to that in clinical samples in the same residential area. The “baseline signal” of EVD68 RNA circulation is 0–4 copies per reaction (Cq values of 38–40), which is around the assay detection limit. During the study period, the number of copies per reaction reached a value of 900 (Figure 1). Importantly, the number of positive cases inevitably derived from the total number of tested cases. Therefore, the actual number of positive cases was potentially higher than what we identified in this study. This potential bias may account for the 2 week difference between the apparent peak in clinically positive cases and the viral load peak in wastewater samples (Figure 1). The actual peak may have been different, but it could not be established with the number of clinical cases examined in this study. Previous reports describe a positive correlation between EVD68 identification in wastewater and clinical samples; in 2014, we first detected EVD68 in sewage samples collected from the residential area of patients positive for EVD68 [10]. A similarly positive correlation between clinical and sewage samples was reported in France during 2014–2015 [8]. More recently, deep sequencing-based identification of an upsurge in EV-D68 circulation in wastewater was reported in the United Kingdom, between July and November 2021 [9]. None of these studies showed a direct quantitative correlation, as we reported herein. Furthermore, deep sequencing is far more complicated and expensive to perform than quantitative PCR and is, therefore, less applicable for national-scale continuous surveillance.

Sequence analysis of partial VP1 gene from positive EV-D68 samples obtained in this study revealed that all samples were of subclade B3 and were almost identical. This finding suggests that all positive cases originated from a single introduction into Israel. This conclusion is in agreement with recent reports that describe the detection of this subclade in additional countries worldwide [25]. The Sanger sequencing procedure identified the dominant strain in the samples. It is possible that by using deep sequencing and library amplification, detection of rare EVD-68 clades can be accomplished. Wide-scale retrospective and prospective EVD68 studies will show whether other clades have emerged in Israel.

In this study, we demonstrated for the first time that quantitative PCR-based EVD68 detection in sewage samples can be used to identify unusual EVD68 circulation and serve as an early warning system for EVD68 outbreaks. The WBE approach, by its nature, is based on pooled samples. Therefore, a relatively small number of WW samples can provide an overall view of viral circulation that is comparable to monitoring thousands of individual clinical samples. Using WW samples is particularly challenging, compared to clinical swab samples, due to the low concentration of viral RNA [26] and high concentration of reaction inhibitors [27]. However, recently developed pre-extraction procedures significantly reduce the presence of inhibitors while concentrating the target RNA, thereby improving the WW-based test sensitivity [28,29]. This approach has been used successfully since the onset of the COVID-19 pandemic for surveillance and epidemiological studies of SARS-CoV-2 [6]. It should be noted that this approach might be biased by rain effects in wastewater systems that collect runoff water. However, this was not the case in this study. The current study reinforces the usefulness of WBE as an important tool to promote the “one health” vision as an early warning system for the emergence and unusual circulation of EVD68 and other viral pathogens. This study was limited to a small (although densely populated) region and a short time period. Expanding this study to encompass a larger geographic area and longer sampling period can be expected to strengthen our findings and add more information on viral presence and load, especially with respect to the onset of unusually increased circulation.

## 5. Conclusions

Wastewater-based surveillance by quantitative RT-PCR may be used as a complementary tool for continuous monitoring of Enterovirus D68 circulation, in parallel with testing of suspicious clinical cases.

## Figures and Tables

**Table 1:** Details of the EVD68-positive clinical cases detected in this study. M—male, F—female, Y—yes, N—no.

Case No.	Sample Date	Age (Years)	Gender	Pre-Existing Disease (Asthma or Other)	Fever	Enteric Symptoms	Respiratory Symptoms	Neurological Symptoms	Dermatological Symptoms	Co-Infection
1	23 May 2021	6	M	Y (Other)	Y	N	Cough, shortness of breath	N	N	N
2	5 May 2021	2	F	Y	N	N	Asthma exacerbation	N	N	N
3	13 June 2021	2	M	Y (Asthma)	Y	N	Rhinorrhea, asthma exacerbation	N	N	N
4	13 June 2021	3	M	N	N	N	Rhinorrhea, asthma exacerbation	N	N	N
5	13 June 2021	3	M	Y (Asthma)	Y	N	Asthma exacerbation	N	N	N
6	13 June 2021	2	F	Y (Asthma)	Y	N	Asthma exacerbation	N	N	N
7	14 June 2021	14	M	Y (Other)	Y	N	Sore throat, weakness, tonsilitis, asthma exacerbation	N	N	N
8	16 June 2021	0	M	Y (Other)	Y	N	N	Aseptic meningitis	N	N
9	17 June 2021	0	M	Y (Other)	Y	N	Respiratory distress	N	N	MSSA
10	17 June 2021	0	M	N	Y	N	Stridor, bronchiolitis, asthma exacerbation	N	N	Adeno in stool
11	25 June 2021	3	M	Y (Other)	Y	N	Rhinorrhea	N	N	GAS mastoiditis
12	27 June 2021	0	M	Y (Asthma)	N	N	Asthma exacerbation	N	N	N
13	27 June 2021	3	F	N	N	N	Asthma exacerbation	N	N	N
14	27 June 2021	7	F	N	N	N	Asthma exacerbation	N	N	N
15	28 June 2021	3	M	Y (Other)	Y	N	Rhinorrhea	N	N	N
16	29 June 2021	3	F	Y (Other)	N	N	Cough, rhinorrhea	N	N	N
17	29 June 2021	0	F	N	N	N	Cough, rhinorrhea	Y	N	N
18	1 July 2021	6	F	Y (Other)	Y	N	Asthma exacerbation	N	N	N
19	5 July 2021	3	F	Y (Other)	N	N	Asthma exacerbation	N	N	N
20	6 July 2021	0	M	N	Y	N	N	Viral encephalitis	N	HHV6 positive in CSF
21	6 July 2021	6	M	N	Y	N	Throat pain, asthma exacerbation	N	N	N
22	8 July 2021	0	F	Y (Other)	Y	N	Cough, rhinorrhea	N	N	N
23	8 July 2021	0	F	N	N	N	N	N	N	N
24	8 July 2021	2	M	N	Y	N	Cough, rhinorrhea; Tonsilitis	N	N	rt pleuropneumonia
25	11 July 2021	1	M	Y (Asthma)	Y	N	Asthma exacerbation	N	N	N
26	12 July 2021	1	M	N	N	N	Rhinorrhea	N	N	N
27	13 July 2021	2	M	Y (Asthma)	Y	N	Asthma exacerbation	N	N	N
28	13 July 2021	3	F	Y (Asthma)	N	N	Asthma exacerbation	N	N	N
29	15 July 2021	2	F	Y (Other)	N	intussusception	ARDS	N	N	N
30	25 July 2021	3	F	N	Y	N	Weakness, asthma exacerbation	N	N	N
31	1 August 2021	1	M	Y (Other)	Y	N	Asthma exacerbation	N	N	N
32	1 August 2021	3	F	Y (Asthma)	Y	N	Asthma exacerbation	N	N	N
33	10 October 2021	2	M	Y (Other)	N	N	N	N	N	N

## Data Availability

Raw data can be provided from the authors upon request.

## Supplementary Materials

The following supporting information can be downloaded at: https://www.mdpi.com/article/10.3390/v14051010/s1, Figure S1: Calibration of the EVD68 − MS2 duplex assay; Table S1: EVD68 + MS2 duplex assay setup for clinical tests; Table S2: EVD68 + MS2 duplex assay setup for wastewater tests; Table S3: Reaction setup for control standard In vitro transcription.
