# Supplementary material for: Monitoring of Enterovirus D68 Outbreak in Israel by a Parallel Clinical and Wastewater Based Surveillance
Source: Viruses. 2022 May 9;14(5):1010. doi: 10.3390/v14051010 (PMC9144596; doi:10.3390/v14051010)
Supplement: Supplementary file 1 [file viruses-14-01010-s001.zip › viruses-1700680-supplementary.pdf]

viruses MDPI

Supplementary Material

Table S1. EVD68+MS2 duplex assay setup and thermal profile for clinical testing.

Component	Final Concentration	Volume/ Reaction(μl)
2X Sensifast Probe Lo-ROX One step		10.00
H_2O		3.30
EVD68 NU Fwd 40μM	600nM	0.30
EVD68 NU Rev1 40 μM	400nM	0.30
EVD68 NU Rev2 40 μM	400nM	0.30
EVD68 NU Probe FAM 20 μM	400nM	0.40
MS2-F 80μM	600nM	0.15
MS2-R 80μM	600nM	0.15
MS2-P 60μM	400nM	0.13
RT-Enzyme SensiFast		0.20
RNAse inhibitor		0.20
RNA		5
Total reaction volume		20

**Thermal Profile**		
**Time**	**Temperature °C**	**Repeats**
15 min	45°C	1
2 min	95°C	1
15 sec	95°C	45
45 sec[1]	55°C	45

[1]Fluorescence was read at the end of each cycle.

Table S2. EVD68+MS2 duplex assay setup and thermal profile for wastewater testing.

Component	Final Concentration	Volume/ Reaction (μl)
4X Bio Rad Reliance mix		5.0
H_2O		8.6
EVD68 NU Fwd 40uM	600nM	0.3
EVD68 NU Rev1 40uM	600nM	0.3
EVD68 NU Rev2 40uM	600nM	0.3
EVD68 NU Probe FAM 20uM	400nM	0.2
MS2-F 80μM	600nM	0.12
MS2-R 80μM	600nM	0.12
MS2-P 60μM	400nM	0.10
Total		15.0
RNA		5

**Thermal Profile**		
**Time**	**Temperature °C**	**Repeats**
15 min	50°C	1
10 min	95°C	1
10 sec	95°C	45
40 sec[1]	55°C	45

[1]Fluorescence was read at the end of each cycle.

Table S3. Reaction setup for control standard *In vitro* transcription.

Component	Volume/Reaction (µl)
2X PCRBIO 1-Step Go mix (www.pcrbio.com/)	20
EVD68 2360Fwd 10µM	1.5
EVD68 2985Rev 10µM stock	1.5
RT enzyme	0.2
H_2O	11.8
RNA	5
Total	40

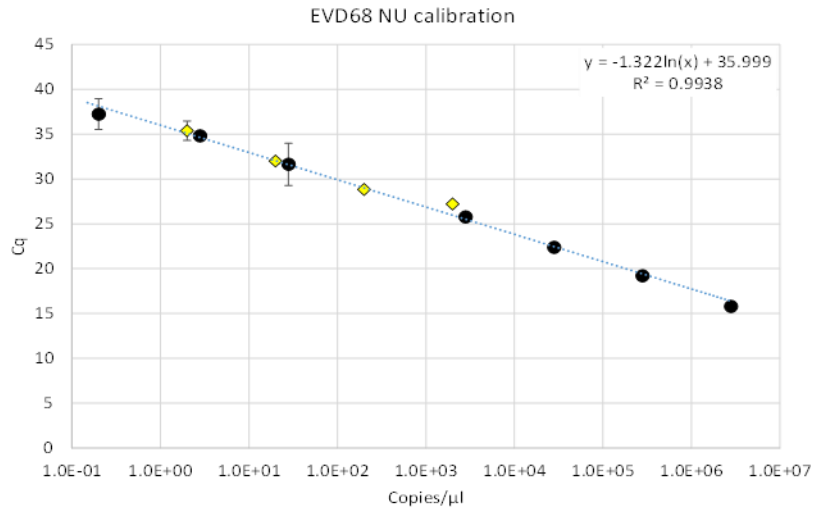

Figure S1. Calibration of the EVD68-MS2 duplex assay. Serial dilutions of IVT standard (black circles) and of commercial whole genome (yellow diamonds), were tested in triplicates using the NU EVD and MS2 primer sets. The standard deviation bars, logarithmic regression formula derived from the calibration curve, and R2 value are shown.
